# P16 Oxazolidinone resistance-associated genes *cfr* and *optrA* in MDR CoNS from healthy pigs in Italy

**DOI:** 10.1093/jacamr/dlac004.015

**Published:** 2022-02-16

**Authors:** Miryam Bonvegna, Elena Grego, Laura Tomassone

**Affiliations:** Veterinary Sciences Department, University of Turin, Largo P. Braccini 2, 10095, Grugliasco, Turin, Italy; Veterinary Sciences Department, University of Turin, Largo P. Braccini 2, 10095, Grugliasco, Turin, Italy; Veterinary Sciences Department, University of Turin, Largo P. Braccini 2, 10095, Grugliasco, Turin, Italy

## Abstract

**Background:**

Oxazolidinones are relatively novel antibiotics used exclusively in human medicine as last resort drugs for resistant pathogens like MRSA, vancomycin-resistant enterococci and penicillin-resistant *Streptococcus pneumoniae.* However*,* in the last two decades, oxazolidinone resistance genes *cfr* and *optrA* have been sporadically reported worldwide in *Staphylococcus* spp. of livestock origin.^1^ These genes can be chromosomal, but they are often transferable through mobile genetic elements, especially plasmids.^2^ In Italy, they have been recently detected in enterococci of swine origin.^3^

**Objectives:**

To uncover the presence of *cfr* and *optrA* genes in methicillin-resistant CoNS (MRCoNS) originating from swine nasal swabs sampled in a high farm-density area of northwestern Italy. Healthy pigs were sampled from three productive stages (finishing, weaners and sows). After isolating pure cultures, selected staphylococci (*n = *27), resulted methicillin-resistant from previous *mecA* identification, were phenotypically tested through Kirby–Bauer disc diffusion method for the antibiotics clindamycin, doxycycline, erythromycin, enrofloxacin, florfenicol, gentamicin, linezolid, tetracycline, tiamulin and trimethoprim/sulfamethoxazole (EUCAST v.11.0 guidelines for linezolid disc, CLSI VET08 for the other antibiotics). MIC through Etest (Liofilchem®, Roseto degli Abbruzzi, Teramo, Italy) was used for the antibiotic ceftaroline.

**Results:**

All the chosen MRCoNS were MDR (MDR CoNS), as they were phenotypically resistant to more than three antibiotic classes. No strain was positive for ceftaroline resistance. Since linezolid resistance was recovered in six samples, we decided to perform PCR for the *cfr* gene (746 bp), which was detected in *Staphylococcus sciuri* from a piglet (GenBank accession number OL412394), and *optrA* (1395 bp), which was recovered in *Staphylococcus pasteuri* from a finisher, *S. sciuri* from a sow and *Staphylococcus cohnii* from a weaner (GenBank accession numbers OM165030, OM165031 and OM165032). Sanger sequencing confirmed PCR result for *cfr*, with 100% identity with the *cfr* gene detected from a clinical Italian isolate of MRSA (MH746818), and for *optrA* gene, which had 100% identity with the *optrA* previously found in a swine Italian *Enterococcus faecium* strain (MT723958). As far as we know, this is the first time that a *cfr* gene has been detected in *S. sciuri* from a nasal sample of animal origin in Italy. Furthermore, *optrA* was never detected in *S. pasteuri* and *S. cohnii* strains.

**Conclusions:**

These results are relevant from a One Health perspective, as they underline the need for oxazolidinone resistance monitoring, not only in human medicine, but also at farm level. In this way, it will be easier to prevent the dissemination of this resistance to human community and hospitals, where oxazolidinones are considered last-resort antibiotics. Furthermore, they remind the importance of surveillance of antibiotic usage in pigs, as *cfr* and *optrA* resistance in staphylococci can be elicited using certain antibiotics, like phenicols, due to cross-resistance to this antibiotic class.
